# Prodrug converting enzyme gene delivery by *L. monocytogenes*

**DOI:** 10.1186/1471-2407-8-94

**Published:** 2008-04-10

**Authors:** Jochen Stritzker, Sabine Pilgrim, Aladar A Szalay, Werner Goebel

**Affiliations:** 1 Biocenter (Microbiology), University of Würzburg, Am Hubland, D-97074 Würzburg, Germany; 2 Genelux Corporation, San Diego Science Center, San Diego, USA; Institute for Biochemistry, Biocenter and Institute for Molecular Infectious Biology, University of Würzburg, Würzburg, Germany; 3 Dade Berhing Marburg GmbH – A Siemens Company, Marburg, Germany; 4 Virchow Center for Biomedical Research, School of Medicine, University of Würzburg, Würzburg, Germany; 5 Max von Pettenkofer Institute for Hygiene and Medical Microbiology, Ludwig Maximillians University, Munich, Germany

## Abstract

**Background:**

*Listeria monocytogenes *is a highly versatile bacterial carrier system for introducing protein, DNA and RNA into mammalian cells. The delivery of tumor antigens with the help of this carrier into tumor-bearing animals has been successfully carried out previously and it was recently reported that *L. monocytogenes *is able to colonize and replicate within solid tumors after local or even systemic injection.

**Methods:**

Here we report on the delivery of two prodrug converting enzymes, purine-deoxynucleoside phosphorylase (PNP) and a fusion protein consisting of yeast cytosine deaminase and uracil phosphoribosyl transferase (FCU1) into cancer cells in culture by *L. monocytogenes*. Transfer of the prodrug converting enzymes was achieved by bacterium mediated transfer of eukaryotic expression plasmids or by secretion of the proteins directly into the host cell cytosol by the infecting bacteria.

**Results:**

The results indicate that conversion of appropriate prodrugs to toxic drugs in the cancer cells occured after both procedures although *L. monocytogenes*-mediated bactofection proved to be more efficient than enzyme secretion 4T1, B16 and COS-1 tumor cells. Exchanging the constitutively P_CMV_-promoter with the melanoma specific P_4xTETP_-promoter resulted in melanoma cell-specific expression of the prodrug converting enzymes but reduced the efficiencies.

**Conclusion:**

These experiments open the way for bacterium mediated tumor specific activation of prodrugs in live animals with tumors.

## Background

Cancer remains one of the most deadly diseases worldwide with the number of cases – especially of melanoma – steadily increasing [1]. Melanoma can be treated by surgical removal at early stages, but once tumor cells have disseminated the current medical therapies are rather ineffective. New approaches for anti-cancer therapy are therefore needed. Among the most promising, more recent developments are the gene directed enzyme prodrug therapies (GDEPT) [2].

All GDEPT operate with the same basic concept: a non-toxic prodrug is converted into a toxic drug inside of cells which were transformed with a gene construct encoding the enzyme needed for the prodrug-drug conversion. For the complete curing of a tumor, transfer of this gene must occur either into all tumor cells or a transformed cell does release enough of the toxic drug to kill the surrounding non-transformed cells. Such "bystander effect" was described for a number of prodrug/drug systems, including the conversions of 5-Fluorocytosine (5-FC) by cytosine deaminase [3], that of 6-Methylpurine-deoxyriboside (MePdR) by the purine nucleoside phosphorylase (PNP) [4-6] or of Fludarabine by the same enzyme [4,5,7,8]. It is crucial that a prodrug-drug converting enzyme is expressed in tumors only. Several strategies were developed to reach this specificity which include the different tumor inoculation methods (reviewed in [9]), conjugate antibody systems directed to specific cell surface antigens, and application of tissue-specific promoters to control the expression of the prodrug converting enzyme (reviewed in [10]).

Tumor-specific promoters have been described for several tumor models especially melanoma [11-16]. One such promoter was constructed by fusing four copies of a mouse tyrosinase enhancer element (TE) to the human tyrosinase promoter (TP). The resulting synthetic promoter P_4xTETP _was shown to be preferentially expressed in tyrosinase-expressing melanomas [15].

*L. monocytogenes *is a facultative intracellular bacterium that replicates efficiently in the cytosol of a wide range of mammalian cells [17]. This microorganism was shown to be an highly versatile carrier for delivering protein antigens, including tumor antigens, directly into the cytosol of infected cells thereby elicting protective cellular immune responses against these antigens (for review see [18]). Virulence-attenuated strains of *L. monocytogenes *were also used to deliver eucaryotic expression plasmids into mammalian cells [19-23]. Furthermore, it was recently reported that *L. monocytogenes *is able to colonize and replicate within solid tumors after local [24,25] or even systemic injection [26].

In this report, we applied *L. monocytogenes*-mediated protein- and DNA-delivery strategies to introduce the prodrug-drug converting enzymes, purine nucleoside phosphorylase (PNP) and a fusion protein consisting of yeast cytosine deaminase and uracil phosphoribosyl transferase (FCU1) into tumor cells and compared the efficiency of the tumor cell inhibition by these two approaches in several tumor cell lines. Furthermore, a melanoma specific promoter was used to enhance specificity of the DNA-delivery strategy.

## Methods

### Strains and plasmids

**Figure 1:**
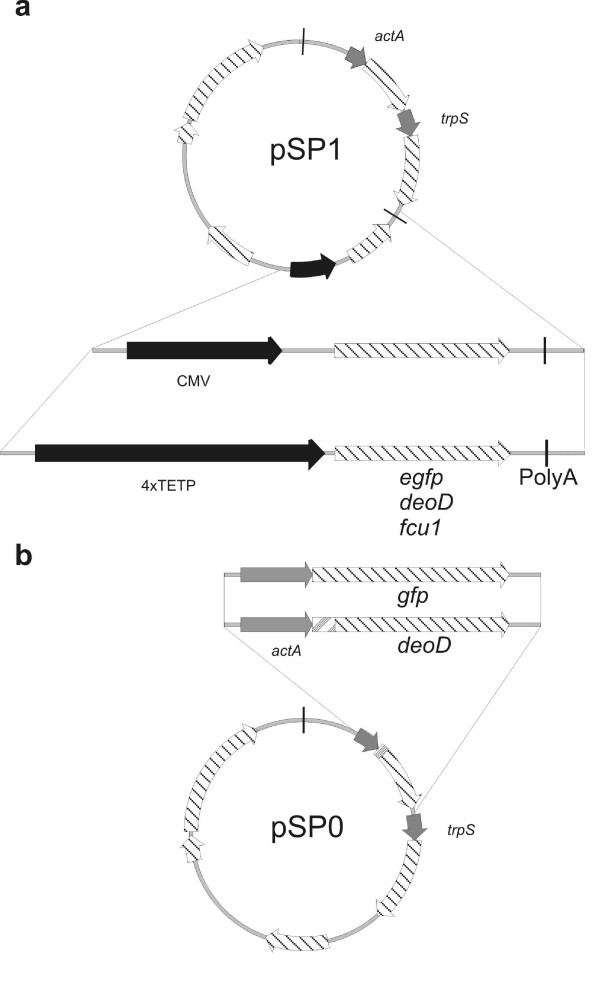
**Construction of the recombinant plasmids used for DNA – (a) and protein-delivery (b)**. Both approaches use the same plasmid backbone consisting of an *E. coli *origin of replication (oriE1), *repD *and *repE *as genes needed for listerial replication, a erythromycin resistance cassette (*ermAM*) and the gene needed for the balanced lethal system *trpS *with its own promoter (P_*trpS*_). (**a**) The genes *egfp*, *deoD*, and *fcu1 *were inserted under the control of the P_CMV _or P_4XTETP _promoters and fused to a poly-adenylation signal. The autolysis cassette consists of the phage lysin gene *ply118 *under the control of the listerial promoter P_*actA*_. (**b**) The *E. coli*-derived gene *deoD *is put under the control of P_*actA *_and genetically fused to the signal sequence (SP) for Sec-dependent secretion of ActA.

*L. monocytogenes *Δ*aroA/B trpS *(WL-150), pTRPS was used as host strain in which pTRPS was replaced by the plasmids described in (Fig. 1). *E. coli *DH10b was used as host for all DNA cloning experiments.

DNA-delivery plasmids were derived from pSP118 [22]. For the construction of pSP118-P_4xTETP_, P_4xTETP _was PCR amplified with primers 4xTETP-PstI-for (5'-ctatcgataggtacc*ctgcag*agttcctgccag-3') and 4xTETP-NotI-rev (5'-cacaag*gcggccgc*gaactggctaattggagtcac-3') using pGL3 4xTETP (kindly provided by S. Hemmi) [15] as template. The 4xTETP-fragment and pSP118 were ligated after cutting with *Pst*I and *Not*I resulting in pSP118-P_4xTETP_. *Egfp *was subcloned from pSP118-P_CMV_-EGFP [22] into pSP118-P_4xTETP _by *Not*I restriction and subsequent ligation from which pSP118-P_4xTETP_-EGFP was obtained. *Fcu1 *was PCR amplified using primers FCU1-NotI-Kozak-for (5'-aaaaaagcggccgctcgccaccatggtgacagggggaatggc-3') and FCU1-NotI-rev (5'-aaaaaagcggccgcttaaacacagtagtatctgtc-3') with pCIneo-*FCU1 *as template [27], thereby introducing *Not*I-sites and a Kozak sequence for efficient translation initiation at the 5'-end of the open reading frame. The resulting fragment was introduced into pSP118 and pSP118-P_4xTETP _using *Not*I restriction resulting in pSP118-P_CMV_-FCU1 and pSP118-P_4xTETP_-FCU1. Plasmids pSP118-P_CMV_-PNP and pSP118-P_4xTETP_-PNP were obtained after PCR amplification of a translation initiation signal from pCMVβ (Clontech) using primers TLI-NotI-for (5'-gtaccc*gcggccgc*aattcccggggatcg-3') and TLI-rev (5'-aatgtgtggggtagccatggtgacttcttttttgct-3') and the open reading frame of *deoD *from *E. coli *chromosomal DNA using primers deoD-for (5'-agcaaaaaagaagtcaccatggctaccccacacatt-3') and deoD-NotI-rev (5'-aaaaaa*gcggccgc*ttactctttatcgcccagc-3') followed by recombinant PCR of the two fragments using TLI-NotI-for and deoD-NotI-rev. The resulting fragment was cloned into pSP118 and pSP118-P_4xTETP _using *Not*I.

For protein delivery *deoD *was amplified using the primers deoD-BamHI-for (5'-aaaaaa*ggatcc*atggctaccccacacattaat-3') and deoD-SacISalI-rev (5'-aaaaaa*cagctggtcgac*ttactctttatcgcccagcag-3') with *E. coli *chromosomal DNA as template. The resulting framgment was cut with *Bam*HI and *Sac*I as was pSP2 P_CMV _[22](thereby removing *ply118*, *trpS *and P_CMV_) and ligation resulted in pUNK P_*actA*-SP_-PNP. The *trpS *gene was again introduced as described previously [22] using the introduced *Sal*I site resulting in the protein delivery plasmid pSP0-P_*actA*-SP_-PNP.

### Cell culture and prodrug treatments

COS-1 (*Cercopithecus aethiops *kidney fibroblasts), 4T1 (murine mammary gland tumor; kindly provided by E. Lukanidin), and B16 (murine skin melanoma; kindly provided by J. Becker) cells were cultured in RPMI 1640 medium supplemented with 2 mM L-glutamine (Gibco, Germany) (RPMI) and 10% fetal calf serum (FCS, Biochrom, Germany), and were maintained at 37°C in a 5% CO_2 _atmosphere.

For prodrug treatment, cells were seeded in 24-well plates 4 days prior prodrug addition. Bactofection was carried out as reported previously [22] and started 3 days before addition of prodrugs. In brief, cells were washed with RPMI, infected for 1 h with a multiplicity of infection (MOI) of 5 bacteria per cell, washed again with RPMI, incubated with gentamicin-containing medium (100 μg/ml) for 1 h which was replaced by medium containing 10 μg/ml gentamicin for subsequent culture.

For protein-delivery, cells were infected with MOI 200 five hours prior to prodrug addition for 1 h. Cells were washed with RPMI and cultivated with gentamicin-containing medium (100 μg/ml) which was replaced with medium containing 10 μg/ml gentamicin after another 1 h. All cells were trypsinized, diluted, and reseeded in 96 well plates (approximately 2 × 10^3 ^cells per well resuspended in 100 μl RPMI containing 10% FCS) 1 h before prodrug addition. Prodrugs were then added in another 100 μl medium resulting in a final concentration of 50 μM for MePdR (Gibco, Germany), 88 μM for Fludarabine (kindly provided by Schering, Germany) and 1 mM for 5-FC (Sigma, Germany) and the prodrug containing medium was left on the cells till the end of the experiment.

### Cell viability assay

The amount of viable cells after prodrug addition was measured using 3-(4,5-dimethylthiazol-2-yl)-2,5-diphenyltetrazolium bromide (MTT, Sigma), following the manufacturer's instructions. At 3, 5, or 7 days after prodrugs were added, medium was replaced by 50 μl MTT solution (2.5 mg/ml MTT dissolved in RPMI without phenol red) and incubated for 4 h at 37°C in a 5% CO_2 _atmosphere. After removal of the MTT solution 1 N HCl diluted in isopropanol was added and the probes were measured at a wavelength of 570 nm. Non-prodrug treated cells were used as reference and were considered as 100% viable.

### HPLC assay of MePdR

HPLC assay was performed as described earlier [14]. In brief, 100 μl cell supernatant was boiled for 15 min and cell debris was removed by centrifuging at 14000 rpm for 15 min at room temperature. Thirty microliters of the supernatant were injected onto a Waters 625 LC-system chromatogroph equipped with a Waters 486 tunable absorbance detector (Waters GmbH, Germany). A Nucleosil 120-5 C_18 _(4 × 250 mm) column (Machery Nagel, Germany) was used with a mobile phase of 50 mM NH_4_H_2_PO_4 _and 90% acetonitrile (Roth, Germany) (flow rate of 1.0 ml/min). MePdR (and MeP) were detected at a UV wavelength of 254 nm, and were identified and quantified by comparing their retention times and absorption spectra with authentic samples.

### Flow cytometry analysis

Three days after infection, bactofected cells were trypsinized and resuspended with PBS. Cell viability was determined by staining cells with propidium iodide (PI, 1.0 μg/ml). Since nonviable cells tend to fluoresce at a similar wavelength as EGFP, PI-positive cells were gated out from the measurement. A minimum of 5 × 10^4 ^cells were then measured using an Epics XL flow cytometer (Beckman Coulter) and data analysis was performed using WinMDI 2.8 (J. Trotter 1993–1998). Cells infected with corresponding strains not encoding EGFP served as negative control. Cells infected for 4 h with WL-150 pSP0-P_*actA*_-GFP were analyzed with the same method.

### Antibody production and Western blot analysis

PNP was overexpressed in *E. coli *M15 using the pQE30 QIAexpress-system (Qiagen, Germany). Therefore, *deoD *was PCR amplified using 6His-BamHI-deoD-for (5'-gataaaggatccgctaccccacacatt-3') and deoD-HindIII-rev (5'-caattaaagcttatcgcccagcagaac-3') with *E. coli *chromosomal DNA as template and cloned into pQE30 using *Bam*HI and *Hind*III restriction sites. 6His-PNP expression was induced by addition of 1 mM IPTG and prepared under native conditions on a Ni-NTA column as described by the manufacturer. Lysis buffer contained 50 mM NaH_2_PO_4_, 300 mM NaCl, and 10 mM imidazole; wash buffer contained 50 mM NaH_2_PO_4_, 300 mM NaCl, and 20 mM imidazole; and elution buffer contained 50 mM NaH_2_PO_4_, 300 mM NaCl, and 250 mM imidazole.

Fifty microgram purified 6His-PNP were suspended in Montanide adjuvans (Merck, Germany) and injected intraperitoneally into CD-1 mice. After 4 weeks, this procedure was repeated and serum was harvested 10 days later.

For Western blot analysis 50 ml of exponentially growing *Listeria*-cultures were centrifuged and the pellet was resuspended in 100 μl/OD_600 _4× Laemmli buffer. Proteins in the supernatant were precipitated on ice for one hour with 10% Trichloroacetic acid. Pelleted proteins were washed with acetone and dissolved in 500 μl 4× Laemmli buffer.

Proteins from mammalian cells were isolated from cells either bactofected for 3 d or infected with the protein-delivery strain for 4 h. Isolation was performed from a 10 cm culture dish using 500 μl RIPA lysis buffer (0.15 M NaCl, 50 mM Tris-HCl, 1% NP-40, 0.5% Deoxycholic acid, 0.1% SDS) with subsequent removal of DNA using sepharose. 15 μl of the supernatant were mixed with 15 μl 4× Laemmli buffer.

After incubation of the protein solution at 98°C for 5 min 30 μl were loaded on a 15% SDS-polyacrylamid gel and electrophoresis was performed by the method of Laemmli [28]. Immunoblotting was performed by a semidry method, with Hybond-ECL nitrocellulose membranes (Amersham Biosciences, Germany). Equal protein load was confirmed by Ponceau S-staining of the nitrocellulose membrane. After incubation with horseraddish peroxidase-conjugated secondary antibodies (Dianova, Germany) and a chemiluminescens-based immunoblot assay (ECL, Amersham Biosciences, Germany) was performed according to the provided instructions.

## Results

### Differential expression of P_4xTETP _in melanoma cells

Different DNA-delivery plasmids were constructed and introduced into an attenuated *L. monocytogenes (Lm) *strain carrying deletions in *aroA*, *aroB *[29], and *trpS *[22] (WL-150). All DNA-delivery plasmids constructed in this study were derived from the recently described vectors pSP118 and pSP0. These plasmids were stabilized by inserting the *trpS *gene (essential for cell viability) into the plasmid and simultaneous deletion of the chromosomal *trpS *copy from the genome. The plasmid pSP118 carried in addition the recently designed autolysis cassette consisting of a phage lysin gene (*ply118*) under the control of the listerial *actA *promoter P_*actA *_[21]. The introduced genes encoding the prodrug/drug converting enzymes used in this approach were placed under the control of the promoter P_CMV _or that of the melanoma-specific P_4xTETP _promoter (Fig. 1a).

**Figure 2:**
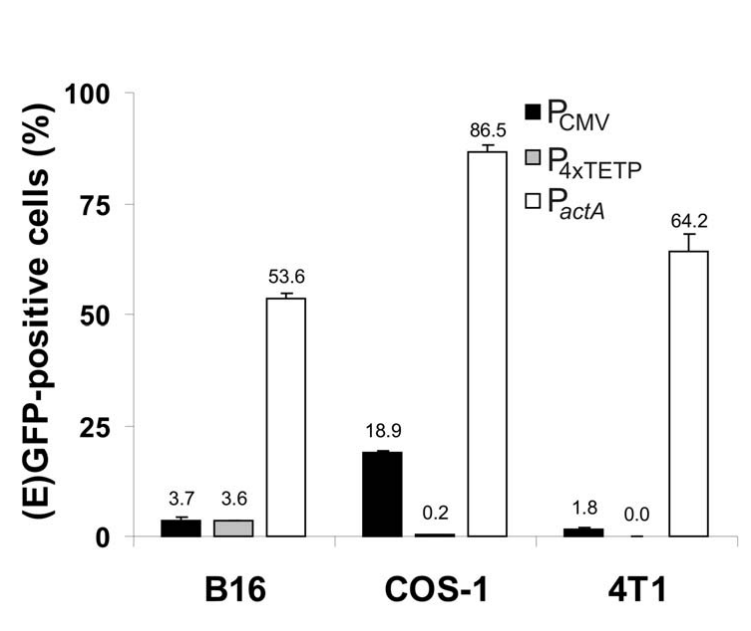
**Determination of the fraction of (E)GFP-positive viable cells**. The cancer cell lines 4T1, B16 and COS-1 were bactofected with WL-150 pSP118-P_CMV_-EGFP (P_CMV_) and WL-150 pSP118-P_4xTETP_-EGFP (P_4xTETP_), respectively, resulting in optimal EGFP production by the bactofected cells after 3 days or these cells were infected with WL-150 pSP0-P_actA_-GFP (P_*actA*_) resulting in GFP synthesis inside the bacteria which was determined 4 h post infection.

For the expression analysis we used COS-1 cells (transformed green African monkey kidney cells) that can be transformed with the *Listeria *as DNA carrier at high rates [22], B16 melanoma cells deriving from a fast growing murine melanoma, and 4T1 cells deriving from a stage 4 breast cancer. These cancer cell lines were transformed at different efficiencies by WL-150 pSP118-P_CMV_-EGFP as determined by flow cytometry of the EGFP-expressing cells (Fig. 2). Transformation with WL-150 pSP118-P_4xTETP_-EGFP carrying *egfp *cDNA under the control of the melanoma-specific P_4xTETP _promoter led to EGFP-expressing B16 melanoma cells at a frequency of 3.6% while COS-1 cells yielded less than 0.3% and 4T1 cells only about 0.04% EGFP-expressing cells (Fig. 2), demonstrating the specificity of the P_4xTETP _promoter for the B16 melanoma cells in the bactofection approach.

### Delivery of prodrug/drug-converting gene constructs to tumor cell lines

**Figure 3:**
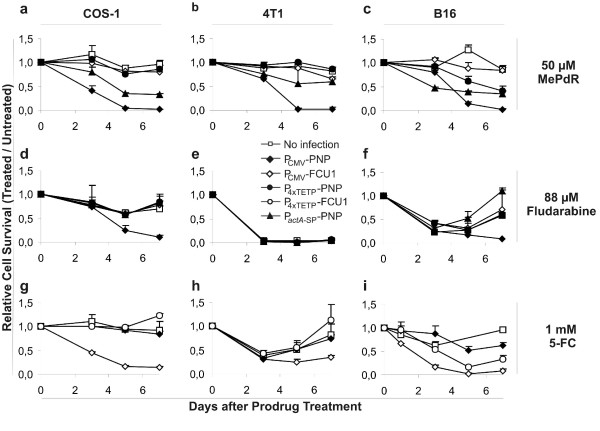
**Inhibition of COS-1- (a, d, g), 4T1- (b, e, h), and B16- (c, f, i) cells bactofected with genes for prodrug-converting enzymes and treated with the indicated prodrug at different time points as result of comparing prodrug-treated versus non-treated cell survival measured by MTT-assay**. The cells were bactofected with WL-150 pSP118-P_CMV_-PNP, WL-150 pSP118-P_CMV_-FCU1, WL-150 pSP118-P_4xTETP_-PNP, and WL-150 pSP118-P_4xTETP_-FCU1, or infected with the protein secreting strain WL-150 pSP0-P_*actA*-SP_-PNP and subsequently treated with 50 μM MePdR (a, b, c), 88 μM Fludarabine (d, e, f), or 1 mM 5-FC (g, h, i); as control un-infected cells were used.

For this study we used two different genes encoding prodrug-drug converting enzymes. Gene *fcu1 *encodes a fusion protein of yeast cytosine deaminase and uracil phosphoribosyl transferase which is highly active in converting of 5-Fluorocytosine (5-FC) into the toxic 5-Fluorouracil and further to 5-Fluoruridine (5-FU) [27]. The gene *deoD *(*E. coli*) encodes a purine-nucleoside phosphorylase (PNP) which converts the prodrug 6-Methylpurine deoxyriboside as well as 9H-Purin-6-amine, 2-fluoro-9-(5-O-phosphono-β-D-arabinofuranosyl) (Fludarabine) to the cell toxic compounds 6-Methylpurine (MeP) and 2-Fluoroadenine (F-Ade) respectively [4]. The expression of the prodrug/drug converting gene constructs (PNP (for MePdR and Fludarabine) and FCU1 (for 5-FC)) were placed under the control of P_CMV _or P_4xTETP_. The efficiency of prodrug-drug conversion was monitored by elimination of cells in the three different cancer cell lines (COS-1, B16 and 4T1) 3 days after DNA delivery. To determine the cell growth the cells were diluted and reseeded in 96 well plates prior to addition of the indicated prodrugs which were added at a final concentration of 50 μM for MePdR, 88 μM for Fludarabine, and 1 mM for 5-FC. The results are shown in Fig. 3(a–i) as the % of viable cells after prodrug treatment compared to untreated cells. Cell viability was measured by the MTT-test.

The data indicate that all three enzymes were functional in COS-1 cells and lead to rapid cell death (Fig. 3a,d,f) when WL-150 was used to introduce the expression cassettes under control of the P_CMV _promoter. COS-1 cells without infection or transformed with *Lm *strains encoding enzymes not able to convert the applied prodrug, consequently were not affected by prodrug addition (Fig. 3a,d,f). In contrast, 4T1 cells were inhibited efficiently and highly specifically by both 5-FC upon infection with WL-150 pSP118-P_CMV_-FCU1 and MePdR when infected with WL-150 pSP118-P_CMV_-PNP. Fludarabine proved to be rather toxic for 4T1 cells and a concentration of 88 μM resulted already in the inhibition of non-infected cells (Fig. 3e). Unspecific obstruction was also observed when using lower concentrations (44 and 30 μM). Less than 15 μM Fludarabine allowed complete survival of the cells but not enough prodrug was converted to the toxic component to affect the 4T1 cells upon infection with WL-150 pSP118-P_CMV_-PNP (data not shown).

In B16 melanoma cells cell-specific expression of PNP and FCU1 was obtained using the melanoma-specific P_4xTETP_-promoter (Fig. 3c,i). However, cell inhibition after treatment of the transformed cells with the prodrugs 5-FC, and MePdR, respectively, was not as efficient as with the same drugs after enzyme production using P_CMV_. Eradication of the entire cell population was obtained with the P_CMV _constructs only (Fig. 3c,f,i).

### Development of *Listeria *as cytosolic protein secretion vector

As alternative to the above described delivery of expression plasmid transfer for prodrug converting enzyme production into cancer cells, we constructed *Lm *strains capable to express and secrete the respective prodrug/drug converting enzyme directly into the host cell cytosol. Therefore, we chose PNP, since FCU1 is a yeast protein and may require posttranslational modification for its function.

**Figure 4:**
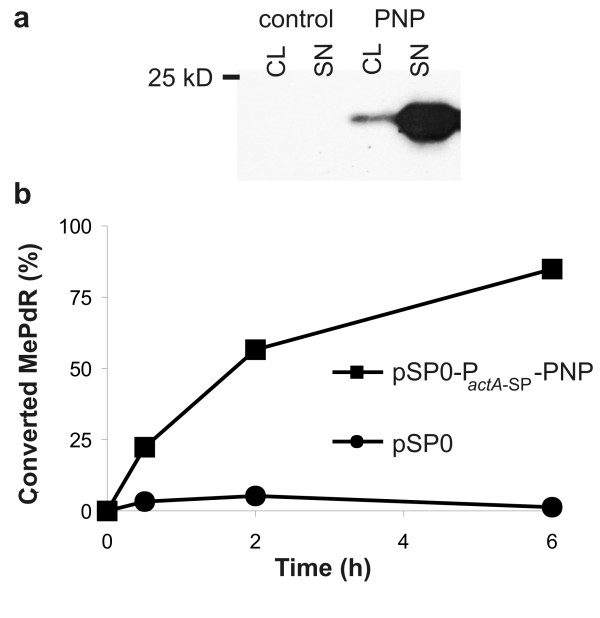
**PNP sectretion and prodrug conversion activity**. Secretion of PNP by WL-150 pSP0-P_actA-SP_-PNP (**a**) The amount of secreted PNP was determined by Western blot analysis with PNP-specific antibodies using listerial cell lysates (CL) or supernatant proteins (SN) obtained by TCA precipitation of cell-free media after growth of WL-150 pSP0 (control) or WL-150 pSP0-P_actA-SP_-PNP (Pnp). (**b**) Prodrug-drug conversion activity of secreted PNP determined by incubation of 100 μM MePdR with the cell-free supernatants from cultures of logarithmically grown WL-150 pSP0 or WL-150 pSP0-P_actA-SP_-PNP at 37°C for up to 6 h. The amount of converted drug was determined by HPLC analysis.

To facilitate PNP secretion from *Listeria *predominantly in the cytosol of the *Lm*-infected cells the *deoD *gene (encoding PNP) was cloned in frame downstream of the promoter and the secretion signal sequence of the listerial *actA *gene. ActA is produced in the cytosol of *Lm*-infected mammalian cells where it is responsible for actin polymerization needed for intra- and intercellular movement of *L. monocytogenes *[30]. The P_*actA*-SP_-PNP cassette was inserted into the recently described *Lm *lethal balanced plasmid pSP0 [31] resulting in pSP0-P_actA-SP_-PNP which was then introduced into WL-150 (Fig. 1b). Western blot analysis of protein preparations from the supernatant and the cell pellet showed that PNP was efficiently secreted by this *Lm *strain (Fig. 4a). Supernatants of logarithmically grown WL-150 pSP0-P_*actA*-SP_-PNP and (as control) WL-150 pSP0 cultured in BHI and supplemented with 1% (w/v) AmberliteTM XAD-4 to enhance P*actA *activity [32] were incubated with 100 μM MePdR for 6 h and conversion to the toxic MeP was determined by HPLC analysis (Fig. 4b). The results showed that 85% of MePdR were converted to MeP by the supernatant of a WL-150 pSP0-P_*actA*-SP_-PNP culture while no conversion was obtained in the control supernatant. Similar results were obtained with lysed bacteria indicating that MePdR can not be converted by the *L. monocytogenes *derived purine deoxyribonucleoside phosphorylases (encoded by *deoD *and *pnp*) annotated in the *Lm *genome [33] (see also ).

After infection of 4T1, B16 and COS-1 cells with WL-150 pSP0-P_*actA*-SP_-PNP at a MOI of 200 for 5 h the prodrug treatment was initiated. Prodrug-dependent inhibition of all cell types was observed when MePdR was added at a final concentration of 50 μM (Fig. 3a–c) to the cultures. However, the addition of 88 μM Fludarabine did not result in specific reduced proliferation of 4T1, B16 or COS-1 cells infected with this *Lm *secretion strain.

### *Listeria *mediated DNA delivery to tumor cells results in efficient prodrug conversion

In a recent study Critchley *et al*. 2004 [34] reported protein-delivery to be more efficient in comparison to DNA-delivery in prodrug therapy using engineered *E. coli *vehicles. The authors based their conclusion on the findings that a higher fraction of GFP expressing cells was found after protein-delivery than after DNA-delivery.

**Figure 5:**
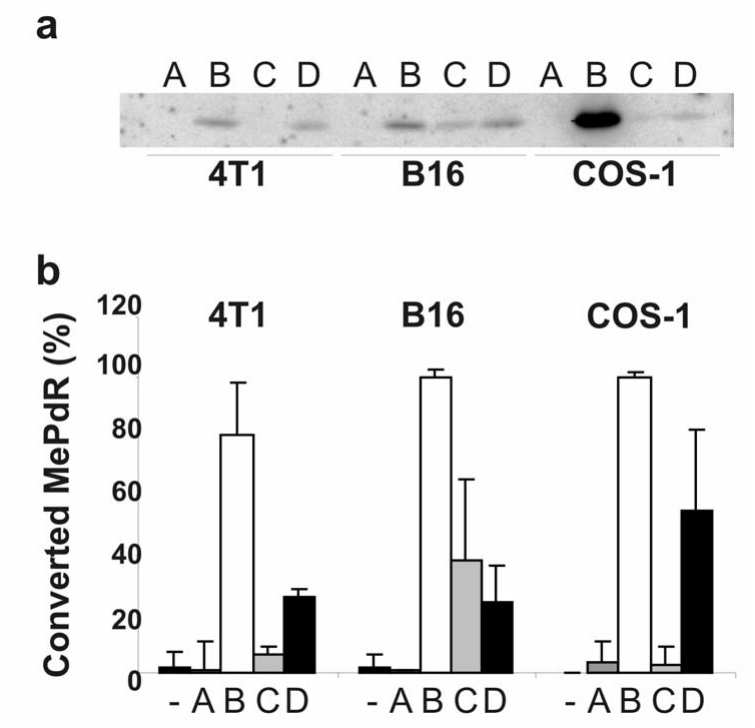
**PNP expression and prodrug-drug conversion after bactofection with the eukaryotic expression plasmids encoding the converting enzymes (genes were under the control of P_CMV _or P_4xTETP_) by COS-1, 4T1 and B16 cells**. **(a) **Western blot analysis with anti-PNP antibodies performed with protein extracts of cells bactofected with WL-150 pSP118-P_CMV_-EGFP (A); WL-150 pSP118-P_CMV_-PNP (B) and WL-150 pSP118-P_4xTETP_-PNP (C) for 3 d or infected with WL-150 pSP0-P_*actA*-SP_-PNP (D) for 4 h. Equal protein load was confirmed by Ponceau S-staining of the nitrocellulose membrane immediately after blotting. **(b) **Conversion of MePdR determined by HPLC-analysis in the supernatant of cell cultures 7 d after addition of 50 μM MePdR.

Our results shown in Fig. 3a–f of this paper suggest, however, a significantly higher conversion efficiency of the prodrug by the enzyme after DNA delivery than after the protein-delivery approach. To determine whether this difference was due to the use of *L. monocytogenes *as bacterial carrier WL150 carrying pSP0-P_*actA*_-GFP encoding GFP under the control of the listerial *actA *promoter was employed as measure of GFP expression inside infected COS-1 cells using a flow cytometer. The results (Fig. 2) showed that the majority of the infected cells indeed did harbour GFP-expressing bacteria similar to the results obtained with the *E. coli *as carrier system. But, although the fraction of cells producing enzymes for prodrug conversion was higher with the protein-delivery approach, the amount of PNP produced in the entire cell populations is higher after DNA delivery (Fig. 5a). Furthermore, densitometrical scanning of the Western blot revealed a 4–6 lesser PNP expression in B16 cells when *deoD *was under the control of P_4xTETP _promoter in contrast to the P_CMV _construct.

In addition, a quantitative comparison of the PNP activity by the two approaches was performed by measuring the amount of the generated toxic compound (MeP). Culture media were taken 7 d after prodrug addition and the non cleaved MePdR concentration was measured by HPLC-analysis. The results shown in Fig. 5b confirmed findings described earlier in Fig. 3 and Fig. 5a.

The reduction of MePdR concentration correlated strictly with the amount of PNP generation. Strain WL-150 pSP118-P_CMV_-PNP as vector resulted in close to 100% conversion of MePdR to MeP in COS-1 and B16 cells, and 80% conversion rate was achieved in 4T1 cells respectively. In contrast, strain WL-150 pSP118-P_4xTETP_-PNP yielded about 40% prodrug conversion in B16 melanoma cells and no conversion was observed in 4T1 or COS-1 cells (Fig. 5b).

The conversion of MePdR after infection of the mammalian cells with PNP-enzyme secreting *Lm *reached only 25–55% in all the cell cultures.

## Discussion

In this work, we report on the construction of plasmids encoding enzymes for prodrug-drug conversion which are carried by a virulence-attenuated *L. monocytogenes *strain. The plasmids are either equipped with eukaryotic expression cassettes encoding these enzymes or with fused genes allowing secretion of the enzymes. In the first case the *Lm *carrier strain releases the expression plasmids into the cytosol of the cancer cells and can reach the nucleus. There, the gene can be expressed and the active enzyme is generated (a procedure for which the term "bactofection" has been coined recently; [22]).

Optimal bactofection frequencies were obtained with a stabilized balanced lethal plasmid system equipped in addition with an autolysis cassette carried by the *Lm *Δ(*aroA trpS*) mutant [22]. The *aroA *mutation provides good virulence attenuation [22,29] and – due to the lack of menaquinone synthesis – leads to a predominantly anaerobic metabolism [29]. For bactofection of the used cancer cells we therefore applied the same plasmid system and the *aroA *mutant strain which carried in addition a deletion in *aroB *to further increase the biosafety of the carrier system [29].

The two prodrug-drug converting enzymes, the *E. coli *purine-nucleoside phosphorylase (PNP) and the yeast hybrid enzyme FCU1, used in this study can either convert MePdR to the toxic compound MeP or Fludarabine to 2-Fluoradenine [35]. FCU1 consists of yeast cytosine deaminase and uracil phosphoribosyltransferase and is also highly active in converting 5-FC to 5-FU [27]. All three prodrug/drug systems were functionally active in the used cancer cell lines (COS-1, B16 and 4T1) (except Fludarabine in 4T1 cells) as shown by the effective inhibition of the *Lm*-infected cells after prodrug treatment compared to the untreated cells.

When the recently described melanoma-specific P_4xTETP _promoter [15] was used instead of P_CMV _for transcribing the genes encoding the prodrug-drug converting enzymes we could observe B16 melanoma specific inhibition but survival of the non-melanoma cell lines COS-1 and 4T1. Therefore, when targeting melanoma, security of the system can be enhanced since gene expression will occur in melanoma cells only.

Among the three prodrugs MePdR, Fludarabine and 5-FC, Fludarabine appears to be least suitable as prodrug in combination with PNP as prodrug-drug converting enzyme. Apart from the rather low conversion rate of Fludarabine to the toxic component by PNP, the non-converted prodrug seems to be already rather cell toxic possibly as a consequence of phosphorylation to the toxic Fludarabine-ATP [36] as can be seen from the growth inhibition in control cells. This seems to be particularly high in 4T1 cells (Fig. 3). *In vivo*, the different sensitivities to Fludarabine could pose a significant problem since treatment of tumor cells (even when expressing the prodrug convering enzyme) might need Fludarabine-concentrations that are toxic to healthy tissues. On the other hand, in CEM cells F-Ade is approximately 100-fold more potent as cytotoxic agent than MeP with a Vmax/Km ratio for the PNP-catalyzed conversion of Fludarabine to F-Ade which is 1/1000 for that of MePdR to MeP [35].

This difference results in the inability of Fludarabine to eradicate COS-1 and B16 cells when these cells were infected with PNP secreting WL-150 or when B16 cells were bactofected with the P_4xTETP_-*deoD *construct.

It is also apparent that 5-FC has some growth-inhibiting effects on 4T1 and B16 cells (control cells in Fig. 3h and 3i), which the cells are able to overcome during the 7 days of incubation. This could be the result of 5-FC instability at 37°C.

Our *in vitro *results show, that the *Lm*-mediated DNA-delivery strategy results in a higher production efficacy of the prodrug converting enzymes in cancer cells and a more efficient inhibition of these cells compared to protein secretion although the fraction of cancer cells in which PNP is expressed is higher with the latter approach. Interestingly, Critchley *et al*. (2004) reported that protein delivery is superior to DNA delivery with *E. coli *as carrier (data confirming this conclusion were based on (E)GFP delivery but no data on treatment of cancer cells with prodrug were provided). The higher enzyme production in the *Lm*-bactofected cancer cells compared to cells infected by *Lm *secreting this enzyme may hence be specific to *Lm *which may not produce the same amount of protein as does *E. coli *under the conditions applied. The sensitivity of *Lm *to the used prodrugs, could also be an explanation for the more efficient prodrug treatment using bactofection compared to protein delivery, however, we did not see any growth defects when the *Lm *strains were coincubated with the prodrugs under investigation. Even when bacteria were grown in the presence of 50 μ M MeP (the active drug) no growth inhibition was observed. In the presence of 1 mM 5-Fluorouracil, we could detect growth inhibition (data not shown), but since 5-FC is no substrate for PNP, this 5-Fluorouracil mediated growth inhibition can not explain the differences detected between PNP-delivery using bactofection and protein delivery, respectively.

As we previously demonstrated several bacteria and viruses replicate highly efficiently in tumor tissue of tumor-bearing mice [25,37] and it was also demonstrated that *Lm *is able to colonize and replicate within tumors [26]. It can therefore be expected that the *Lm-*mediated bactofection system used here for the cell culture studies will also function in live animals. The more anoxic tumor environment may even favour replication of the used *Lm *carrier strain which exhibits a predominantly anaerobic metabolism due to the *aro *mutation [29]. Even an *Lm*-mediated protein-delivery approach could benefit from the tumor specific colonization and replication of the carrier bacteria. Since we do not know at the moment whether *Lm*-mediated delivery of expression plasmids is also superior to protein delivery *in vivo *the latter approach should also be considered for *in vivo *application of the reported *Lm *system, especially when taking into account that protein-delivery was advantageous compared to DNA-delivery in vivo in terms of eliciting immune-responses against heterologous antigens [22]. One could also speculate that the protein delivery approach has advantages over bactofection *in vivo*, because of the autolysis mediated by phage lysin expression. This might inhibit efficient bacterial spreading in tumors, thus also reducing the observed bystander effect. Tumor-specific promoters for the transcription of the genes encoding the prodrug-converting enzymes may not be required as tumor-specific expression of these enzymes may be already guaranteed by the tumor-specific colonization of the carrier bacteria but nevertheless, the security of the system would be improved.

## Conclusion

In summary, protein- as well as particularly DNA-delivery by the reported virulence-attenuated *Lm *carrier may become valuable tools for future tumor therapy. A combination of both strategies may simultaneously deliver tumor antigens and prodrug-drug converting enzymes via the virulence-attenuated *Lm *carrier into a tumor-bearing organism. This approach would thus link bacterial carrier-supported tumor immune therapy with chemotherapy.

## Competing interests

JS, and AAS declare competing financial interest.

## Authors' contributions

JS: conception and design of study; acquisition, analysis and interpretation of data; drafting the manuscript. SP: interpretation of data, critical revision of manuscript. AAS: critical revision of manuscript. WG: concept of study; interpretation of data; drafting of manuscript

## Pre-publication history

The pre-publication history for this paper can be accessed here:


